# DNA microarray data integration by ortholog gene analysis reveals potential molecular mechanisms of estrogen-dependent growth of human uterine fibroids

**DOI:** 10.1186/1472-6874-7-5

**Published:** 2007-04-02

**Authors:** Tao Wei, Andrew G Geiser, Hui-Rong Qian, Chen Su, Leah M Helvering, Nalini H Kulkarini, Jianyong Shou, Mathias N'Cho, Henry U Bryant, Jude E Onyia

**Affiliations:** 1 Integrative Biology, Lilly Research Laboratories, Greenfield, Indiana 46140, USA; 2 Bone and Inflammation, Lilly Research Laboratories, Indianapolis, Indiana 46285, USA; 3 Discovery Statistics, Lilly Research Laboratories, Indianapolis, Indiana 46285, USA

## Abstract

**Background:**

Uterine fibroids or leiomyoma are a common benign smooth muscle tumor. The tumor growth is well known to be estrogen-dependent. However, the molecular mechanisms of its estrogen-dependency is not well understood.

**Methods:**

Differentially expressed genes in human uterine fibroids were either retrieved from published papers or from our own statistical analysis of downloaded array data. Probes for the same genes on different Affymetrix chips were mapped based on probe comparison information provided by Affymetrix. Genes identified by two or three array studies were submitted for ortholog analysis. Human and rat ortholog genes were identified by using ortholog gene databases, HomoloGene and TOGA and were confirmed by synteny analysis with MultiContigView tool in the Ensembl genome browser.

**Results:**

By integrated analysis of three recently published DNA microarray studies with human tissue, thirty-eight genes were found to be differentially expressed in the same direction in fibroid compared to adjacent uterine myometrium by at least two research groups. Among these genes, twelve with rat orthologs were identified as estrogen-regulated from our array study investigating uterine expression in ovariectomized rats treated with estrogen. Functional and pathway analyses of the twelve genes suggested multiple molecular mechanisms for estrogen-dependent cell survival and tumor growth. Firstly, estrogen increased expression of the anti-apoptotic PCP4 gene and suppressed the expression of growth inhibitory receptors PTGER3 and TGFBR2. Secondly, estrogen may antagonize PPARγ signaling, thought to inhibit fibroid growth and survival, at two points in the PPAR pathway: 1) through increased ANXA1 gene expression which can inhibit phospholipase A2 activity and in turn decrease arachidonic acid synthesis, and 2) by decreasing L-PGDS expression which would reduce synthesis of PGJ2, an endogenous ligand for PPARγ. Lastly, estrogen affects retinoic acid (RA) synthesis and mobilization by regulating expression of CRABP2 and ALDH1A1. RA has been shown to play a significant role in the development of uterine fibroids in an animal model.

**Conclusion:**

Integrated analysis of multiple array datasets revealed twelve human and rat ortholog genes that were differentially expressed in human uterine fibroids and transcriptionally responsive to estrogen in the rat uterus. Functional and pathway analysis of these genes suggest multiple potential molecular mechanisms for the poorly understood estrogen-dependent growth of uterine fibroids. Fully understanding the exact molecular interactions among these gene products requires further study to validate their roles in uterine fibroids. This work provides new avenues of study which could influence the future direction of therapeutic intervention for the disease.

## Background

Leiomyoma or uterine fibroids are the most common benign tumor, occurring in approximately 60% of women during their lifetime[1]. In spite of its generally benign nature, uterine fibroids cause an array of substantial health problems in some women such as pressure or pain, excessive uterine bleeding and problems related to pregnancy [2]. As a consequence, uterine fibroids account for approximately one-third of all hysterectomies in the United States or about 200,000 hysterectomies per year [3]

Although the etiology of the disease is largely unknown, it is clear that growth of uterine fibroids depends on the ovarian hormones estrogen and progesterone [2]. This hormonal dependency is supported by the following observations. Uterine fibroids are observed only after menarche, increase in size during pregnancy, and frequently regress after menopause (reviewed in [2]). The tumors can be induced to regress by surgical ovariectomy or by treatment with GnRH agonists which induce a hypoestrogenic state[4]. Tissue estrogen concentrations are elevated in uterine fibroids compared to myometrium, which may result from increased aromatase activity [5]. Estrogen produces diverse biological effects mediated by estrogen receptors (ER). When bound to estrogen, the ER modulates the transcriptional activity of target genes [6,7]. Evidence shows that one effect of estrogen is to increase the levels of both estrogen receptor (ER) and progesterone receptor (PR) [2]. It has been recently demonstrated that estrogen can stabilize ER mRNA, increasing the level of cellular ER protein [8].

While it is well established that growth of uterine fibroids depends on estrogen, molecular mechanisms of such estrogen dependency are largely unknown. Numerous studies have indicated that estrogen may mediate fibroid growth through the mitogenic effects of growth factors such as transforming growth factor-β and basic fibroblast growth factor (reviewed in [2]). There have been a few recent studies addressing molecular mechanisms of functional interaction between estrogen signaling and growth factor-mediated signaling in the pathogenesis of uterine fibroids. Work by Hayashi et al [9] in estrogen-dependent cancers provides an example where the constitutively activated MAPK signaling pathway in endometrial cancer cells might enhance the transcriptional activity of ERα via phosphorylation of its AF-1 domain. Wnt signaling was recently implicated in the pathogenesis of uterine fibroids where the secreted frizzled related protein 1 (sFRP1) mRNA [10] was found to be significantly elevated in the tumor, and regulated by estrogen treatment. It was shown that sFRP1 contributes to fibroid growth through an anti-apoptotic effect.

A recent report has shown that PPARγ activation by its ligand (i.e., prostaglandin J2) in uterine fibroids is growth inhibitory and mediated at least in part by negative cross-talk between ER and PPARγ signaling pathways [11]. However, the exact molecular mechanisms of how such interaction occurs between the two nuclear receptor signaling pathways remain to be answered. Elevated levels of PPARγ, retinoid × receptor alpha (RXRα), and all-trans retinoic acid were observed in human uterine fibroids, and retinoids in combination with estrogen was shown to induce development of uterine fibroids in a guinea pig model [12].

DNA microarray technology allows simultaneous interrogation of tens of thousands of genes [13]. Several studies have applied the technology to identify genes that were differentially expressed in human uterine fibroids compared to adjacent normal myometrium [14]. The technology has also been applied to identify estrogen-regulated genes in the adult rat uterus [15]. To understand the molecular interactions involved in estrogenic support of uterine fibroid growth, we integrated results from the above DNA microarray studies using ortholog analysis. Orthologous genes are related by direct evolutionary descent and should play similar developmental or physiological roles [16]. This study identified twelve human and rat orthologous genes that were differentially expressed in human uterine fibroids and estrogen-regulated in the adult rat uterus, and describes their possible cellular and physiological roles in estrogen-dependent tumor growth.

## Methods

### Published DNA microarray data sets

Table 1 lists four published DNA microarray data sets used in this work. Briefly, researchers [14,17,18] profiled biopsy samples taken from uterine fibroids and myometrial tissues of each patient (paired experimental design) to identify differentially expressed statistically significant (P < 0.05) genes. Helvering et al [15] profiled genes expressed in the uteri of adult ovariectomized rats treated with estrogen for 24 hours or for five weeks. Estrogen regulated genes were identified as genes with false discovery error rates < 0.05 [19] and showed an opposite direction of change when comparing genes differentially expressed between ovariectomized and sham controls versus genes differentially expressed between ovariectomized controls with ovariectomized animals treated with estrogen (E) at either time points

**Table 1:** Published DNA microarray experiments

Species	Tissue	Chip Used	Sample Size	No of Genes Identified	References
Human	Uterine fibroids and normal myometrium	HG-U95A HuGeneFL	9 pairs	106^a^	[14]
		HuGeneFL	7 pairs	68	[17]
		HG-U133A	5 pairs	226^b^	[18]
Rat	Uteri from vehicle and estrogen treated animals	RG-U34A	5 rats per treatment, 2 chip for each animal	3927^c^	[15]

^a ^the authors reported 145 genes but only 106 accessions could be retrieved from the paper.

^b ^represents the number of the Affymetrix probe sets.

^c ^the probe sets were significant with false discovery rate < 0.05 by either one – day or five – week estrogen treatment of ovariectomized rats.

### Identification of human and rat ortholog genes that are estrogen responsive and differentially expressed in uterine fibroids

**Figure 1:**
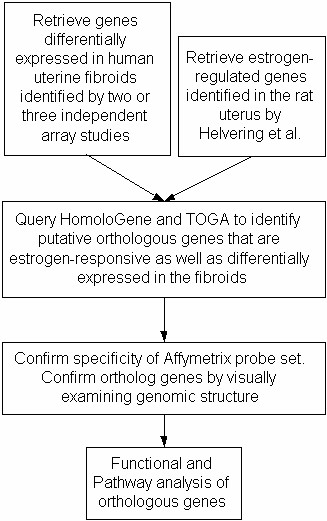
Diagram of DNA microarray data integration by ortholog gene analysis.

Figure 1 shows the procedure that was employed to identify human and rat ortholog genes that were differentially expressed in human uterine fibroids and estrogen-regulated in rat uterus. In the first step two gene lists were constructed. The first gene list included human genes differentially expressed in human uterine fibroids identified by three independent groups [14], which were retrieved from either published papers or from the **G**ene **E**xpression **O**mnibus database [20]. Since the three studies employed distinct Affymetrix chip types the information of chip comparison was retrieved from the NetAffx web site[21] to map genes from one chip type to another. Genes commonly identified by two or three groups were regarded as true differentially expressed genes and thus selected for ortholog analysis. The second gene list included estrogen-responsive genes in the rat uterus which were obtained from the work by Helvering et al[15]. The second step was to identify putative ortholog genes from these two gene lists. Two major eukaryotic ortholog gene databases, namely HomoloGene [22] and TOGA [23], were queried for each of the tumor differentially expressed genes to identify putative rat orthologs and LocusLink numbers were then used to retrieve Affymetrix probe sets on rat RG-U34A chips. These probe sets were next compared with probe sets identified by Helvering et al [15] to find putative human rat orthologs. Finally, an *in silico *confirmation of expression and ortholog gene identity was performed by examining each Affymetrix probe set for its specificity to measure expression using the BLAT program [24] which aligned Affymetrix's target sequences against both human and rat genomes. Genes were considered if their Affymetrix target sequences mapped to only one major genome location. To confirm orthologous genes identified above, each gene was queried in the Ensembl genome browser [25] and gene structure, chromosomal location and synteny information were visually examined with Ensembl MultiContigView [25]. Lastly, functional and pathway analysis of ortholog genes were conducted to understand cellular and physiological implications.

To calculate functional distribution of the human genes differentially expressed in uterine fibroids, a web tool FatiGO developed by [26] was used according to its on-line instruction.

## Results

### Genes differentially expressed in human uterine fibroids

**Figure 2:**
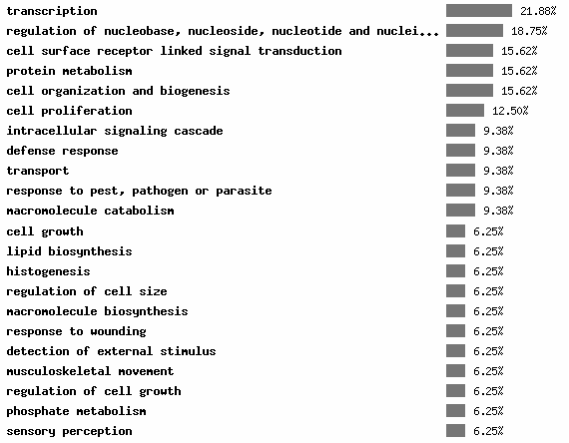
Functional distribution of thirty-eight genes differentially expressed in human uterine fibroids. The analysis was done using a web tool FatiGO developed by Al-Shahrour et al. [26]. Since some genes are involved in multiple biological processes the sum of percentage is more than 100.

Differential expression of genes between human uterine fibroids and adjacent normal myometrium were identified by three independent research groups as shown in Table 1. The number of genes identified was significantly different among the three studies. All three studies used matched fibroid and myometrial tissue. However, the studies differed in the following aspects. First, the ethnic background of the patients was very different. For example, in Wang's experiment the patients were largely African-American whereas in the Tsibris et al study the patients were mainly of Northern European descent. It is well known that uterine fibroids are much more prevalent in African-American women than in Caucasian women (reviewed in [2]). Secondly, tissues were sampled from patients at different ovarian phases. The number of genes identified was significantly greater in the Hoffman and Tsibris studies where samples were taken from both the follicular and luteal phases compared to the Wang study where the authors sampled tissues from the follicular phase only. Thirdly, different DNA chips were used (Table 1). These differed in the number of genes arrayed on each chip, probe pairs for a given probe set, and probe pairs for the same gene that might be taken from different metrix target sequences. By using information on chip comparisons provided by NetAffx[21], we identified thirty-eight genes (Table 2) that were commonly reported to be significantly changed in the same direction in two or three studies. Functional distribution within gene ontology space (Figure 2) was calculated by the FatiGO program [26]. The top ranked functional categories include transcription, regulation of nucleotide metabolism, cell surface receptor-linked signal transduction, protein metabolism, cell organization and biogenesis, cell proliferation, intracellular signaling cascade, defense response and transport.

**Table 2:** Genes with differential expression in human uterine fibroids identified by two or three groups of researchers.

Accession^a^	Symbol^b^	Gene Descritpion^b^	H^c^	T^c^	W^c^
NM_007168	ABCA8	ATP-binding cassette, sub-family A (ABC1), member 8	-2.5	-5.7	
NM_006720	ABLIM1	actin binding LIM protein 1	-3.3	-4.28	
NM_000689	ALDH1A1	aldehyde dehydrogenase 1 family, member A1	-3.5	-7.98	-4
NM_000700	ANXA1	annexin A1	-4.1	-4.32	
NM_001674	ATF3	activating transcription factor 3	-8.8	-6.04	
M18767	C1S	complement component 1, s subcomponent	-2.5		-3.2
NM_004772	C5orf13	chromosome 5 open reading frame 13	2.45	2.81	
NM_001218	CA12	carbonic anhydrase XII	3.81	4.8	
U17473	CALCRL	calcitonin receptor-like receptor	-2.1		-1.7
NM_001870	CPA3	carboxypeptidase A3 (mast cell)	-2.1	-8.18	
NM_001878	CRABP2	cellular retinoic acid binding protein 2	3.05	5.12	2.6
AF003114	CYR61	cysteine-rich, angiogenic inducer, 61	-5.8	-5.3	
AI826799	EFEMP1	EGF-containing fibulin-like extracellular matrix protein 1	-4.1	-4.51	-5
BC004490	FOS	v-fos FBJ murine osteosarcoma viral oncogene homolog	-9.5	-8.17	
NM_007003	GAGEC1	G antigen, family C, 1	2.98	40.7	
NM_000826	GRIA2	glutamate receptor, ionotropic, AMPA 2	7.48	38.8	4.9
J03242	IGF2	insulin-like growth factor 2 (somatomedin A)		16.8	2.6
NM_002178	IGFBP6	insulin-like growth factor binding protein 6	-4.5	-5.26	
NM_004522	KIF5C	kinesin family member 5C	2.12	9.02	
AL021977	MAFF	v-maf musculoaponeurotic fibrosarcoma oncogene homolog F (avian)	-6.8	-3.38	
NM_005923	MAP3K5	mitogen-activated protein kinase kinase kinase 5	-2.1	-4.13	-3
NM_002402	MEST	mesoderm specific transcript homolog (mouse)	3.87	11.6	2.4
NM_002135	NR4A1	nuclear receptor subfamily 4, group A, member 1	-3.7	-3.78	
NM_006198	PCP4	Purkinje cell protein 4	4.85	8.23	
AI934473	PIK3R1	phosphoinositide-3-kinase, regulatory subunit, polypeptide 1 (p85 alpha)	3.01	2.21	
BC002665	PLP1	proteolipid protein 1 (Pelizaeus-Merzbacher disease, spastic paraplegia 2, uncomplicated)	4.75	12.1	
BC005939	PTGDS	prostaglandin D2 synthase 21kDa (brain)	-2	-3.47	-2
D86096	PTGER3	prostaglandin E receptor 3 (subtype EP3)		-4.2	-2.8
NM_002937	RNASE4	ribonuclease, RNase A family, 4	-2	-3.38	
U89281	RODH	3-hydroxysteroid epimerase	1.93	3.34	
NM_000602	SERPINE1	serine (or cysteine) proteinase inhibitor, clade E, member 1	-4.4	-4.09	
BG528420	SOX4	SRY (sex determining region Y)-box 4	3.98	2.81	
NM_003242	TGFBR2	transforming growth factor, beta receptor II	-1.8	-2.02	
NM_021992	TMSNB	thymosin, beta, identified in neuroblastoma cells	1.59	5.51	
NM_007116	TNXB	tenascin XB	-2.2	-6.28	
NM_003294	TPSB2	tryptase beta 2	-4.1	-24.3	
AF349719	TRO	trophinin	1.74	3.3	
NM_001071	TYMS	thymidylate synthetase	2.1	9.12	3.3

^a ^Genbank or RefSeq accession number.

^b ^Gene symbol or description were obtained from the BioConductor Project.

^c ^Fold change of gene expression in uterine fibroids vs myometrium. H, T and W represent studies by Hoffman et al. [18], Tsibris et al. [14] and Wang et al. [17] respectively.

### Estrogen-regulated genes in the uterus of ovariectomized adult rats

Helvering et al [15] evaluated short and long-term effects of ovariectomy and treatment with estrogen on expression changes in the uterus of ovariectomized rats. They found that ovariectomy induced dramatic gene expression changes in the uterus both at 13 days and at 5 weeks post surgery with 1930 or 2908 Affymetrix probes changed, respectively. Treatment of ovariectomized rats with 0.1 mg/kg/day 17-β ethinyl estradiol also induced significant changes with 2389 probes altered following 1-day of treatment and 2990 probes following 5-week treatment. In total, there were 3927 Affymetrix probe sets that were changed by ovariectomy and then altered in the opposite direction by estrogen treatment of ovariectomized rats at two time points.

### Identification of orthologous genes differentially expressed in human uterine fibroids and regulated by estrogen in the rat uterus

Each of the thirty-eight genes differentially expressed in human uterine fibroids was queried against the HomoloGene and TOGA databases for rat ortholog genes, among which twelve genes were found to be responsive to estrogen treatment in the rat uterus. Table 3 shows relative changes of expression of the twelve human and rat orthologs obtained from the original study. All twelve genes showed high and consistent transcriptional changes in uterine fibroids across two or three independent studies and also responded to ovariectomy and estrogen treatment in reversed manner consistently at both time points.

**Table 3:** Human and rat orthologous genes differentially expressed in human uterine fibroids and E2-responsive in the rat uterus.

**Gene**	**Symbol**	**Uterine fibroids**			**1-Day**		**5-Week**		**Previous reports on E2 regulation**
			
		**H**^a^	**T**^a^	**W**^a^	**ovx**	**E2**	**ovx**	**E2**	
prostaglandin E receptor 3 (subtype EP3)	PTGER3		-4.2	-2.8	1.8	-1.8	1.6	-1.7	None
transforming growth factor, beta receptor II (70/80kDa)	TGFBR2	-1.8	-2.0		3.3	-3.2	5.4	-15.6	None
Purkinje cell protein 4	PCP4	4.9	8.2		NS	NS	-1.4	2.4	None
calcitonin receptor-like receptor	CALCRL	-2.1		-1.7	1.9	-1.7	1.2	-1.3	[35] [34]
aldehyde dehydrogenase 1 family, member A1	ALDH1A1	-3.5	-8.0	-3.8	7.7	-9.0	4.5	-4.4	[28] [29]
annexin A1	ANXA1	-4.1	-4.3		-1.4	1.6	NS	NS	[33]
cellular retinoic acid binding protein 2	CRABP2	3.1	5.1	2.6	-23.1	37.6	NS	NS	[28] [29]
prostaglandin D2 synthase 21kDa (brain)	L-PTGDS	-2.0	-3.5	-2.1	2.0	-2.3	1.8	-2.0	[30, 31] [32]
nuclear receptor subfamily 4, group A, member 1	NR4A1	-3.7	-3.8		NS	NS	-1.8	5.4	[37] [64]
proteolipid protein 1	PLP1	4.8	12.1		1.3	-1.5	1.3	-1.3	None
ribonuclease, RNase A family, 4	RNASE4	-2.0	-3.4		NS	NS	3.5	-4.3	None
insulin-like growth factor 2 (somatomedin A)	IGF2		16.8	2.6	NS	NS	2.2	-1.6	Contradictory to [38] [40] [39]

^a ^Abbreviations: H for Hoffman et al study [18], T for Tsibris [14], and W for Wang et al. [17], ovx = ovariectomized rats, E2 = 17β – estradiol, and NS = not statistically significant at α = 0.05.

To confirm that the Affymetrix probe sets were indeed specific for each gene we confirmed their alignment with the human and rat genomes using the BLAT tool [24] at UCSC [27]. Except for Affymetrix target sequence of rat gene C1S which produced two major alignments in the q42 cytoband of rat chromosome 4, one spanning 160981068 to 160993069 with 98.6% identities and the other spanning 160609849 to 160612122 with 75.7% identities, Affymetrix target sequences of all the other twelve genes generated one major alignment to the expected gene in the human and rat genomes. For example, the rat target sequence for the rat L-PGDS gene had a single, major alignment with the L-PGDS gene on chromosome 3 in the rat genome and the human target sequence generated a single alignment with L-PGDS gene on chromosome 9 in the human genome. Thus we believe that expression signals of the twelve human and rat orthologs, except for the gene C1S, obtained by Affymetrix chips used in these studies could accurately measure mRNA abundance of the twelve genes in the human and rat samples.

**Figure 3:**
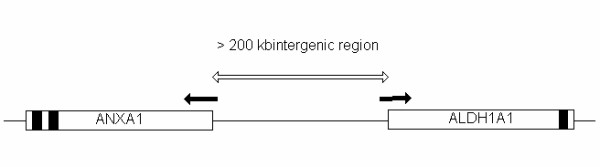
Conserved genomic structure of ALDH1A1 and ANXA1 genes among rat, mouse and human. Both genes are located on the same chromosome with head-to-head configuration. The configuration is conserved among three organisms. Filled arrows indicate transcription direction. Filled black bars are target sequences from which Affymetrix probes were derived. Over 200 kb of intergenic sequence is indicated by an empty arrow.

To demonstrate that the twelve genes are genuinely human and rat ortholog genes, each of them was queried in the UCSF and Ensembl genome browsers to visually examine the local genome structure and compare them between the two species. Figure 3 shows the genome structure of ALDH1A1 and ANXA1 as an example. Interestingly, ALDH1A1 and ANXA1 genes map to a common chromosomal region in both species and the genomic structures are well conserved among human, mouse and rat. This relationship, regarded as a synteny, provides strong evidence for orthologship of the two genes among human, rat and mouse. Similarly, the rest of the ten genes are also located in their respective syntenic chromosomal regions in two species (data not shown).

A literature search was conducted for each of the twelve genes to determine if others have previously identified these genes to be directly regulated by estrogen (Table 3). Cellular retinoic acid binding protein 2 (CRABP2) and aldehyde dehydrogenase 1 A1 (ALDH1A1) were previously shown to directly respond to estrogen in the rat uterus [28,29] in the same fashion as observed by Helvering using DNA microarray. Lipocalin-type prostaglandin D synthase (L-PGDS) was reported to respond to estrogen in a more complicated fashion depending on the tissues/organs. For example, L-PGDS transcription was induced in the medial basal hypothalamus and repressed in the preoptic area in female adult mice by estrogen [30,31]. Transcription of L-PGDS was also induced in mouse heart *in vivo *specifically by estrogen receptor beta via a functional estrogen-responsive element in the L-PGDS promoter [32]. Castro-Caldas et al [33] demonstrated that estrogen induced de novo expression of annexin A1 (ANXA1) and stimulated its secretion in the human CCRF-CEM acute lymphoblastic leukemia cell line apparently via a mechanism independent of the intracellular estrogen receptor. Consistent with this result we couldn't find any putative estrogen responsive element in its 5 kb promoter sequence. Expression of calcitonin receptor-like receptor (CALCRL) was inhibited by estrogen in the rat uterus [34] and placenta [35]. Nuclear receptor subfamily 4, group A, member 1 (NR4A1) whose human promoter harbors a potential estrogen-responsive element (ERE) [36], was reported [37] to be an early responsive gene in the ovarectomized rat uterus to estrogen treatment. Insulin-like growth factor II (IGF2) was regulated in the estrogen-treated rat uterus (Table 3), but had previously been reported to be estrogen non-responsive. Rather, IGF-1 was found to be regulated by estrogen in human uterine fibroids [38], rhesus monkey myometrium [39] and rat uterus [40].

In summary, by integrating multiple independent DNA microarray studies of differentially expressed genes in human uterine fibroids a group of thirty-eight genes were identified as consistently changed in the tumor versus surrounding normal myometrium. By ortholog gene analysis we identified a subset of these genes that were estrogen-regulated in the rat array study. Six of the twelve ortholog genes have previously been described to be regulated by estrogen while the remaining genes have yet to be independently verified as estrogen-responsive.

## Discussion

DNA microarray technology provides us with a great opportunity for looking at molecular mechanisms of disease development on a whole genome scale. While a few public databases have been built to facilitate data sharing [20,41,42], it still remains a great challenge to integrate the data, particularly data generated from different organisms, in order to generate testable hypotheses. In the present work we integrated multiple microarray data sets generated by independent research groups from two different species using ortholog gene analysis to try to discover molecular clues to estrogen-dependent growth of human uterine fibroids. While this *in silico *analysis suggests pathways and individual gene product involvement in the regulation of fibroid tumor growth by estrogenic signaling, the authors recognize the need for experimental follow-up to prove these associations.

Thirty-eight human genes (Table 2) were identified in common from three independent studies showing differential expression between uterine fibroids and normal myometrium. Of these, twelve human and rat orthologous genes (Table 3) were shown to be estrogen-regulated in the rat uterus. Since they are human and rat orthologs, we inferred that they should share similar expression regulation and biological functions in both species. These genes provide important clues to understand estrogen-dependent growth of human uterine fibroids.

Prostaglandin E2 receptor subtype 3 (PTGER3 or EP3) is a G-protein-coupled receptor activated by prostaglandin E2 that was down regulated in uterine fibroids. Alternative splicing generates three isoforms: EP3 alpha, EP3 beta and EP3 gamma, which differ in the putative cytoplasmic carboxy-terminal tail. It was demonstrated that while EP3 gamma is coupled to both stimulation and inhibition of adenylate cyclase, EP3 alpha and beta are exclusively coupled to inhibition of adenylate cyclase [43]. Shoji et al. [44] demonstrated that EP3 plays an important role in suppression of cell growth and its down-regulation enhances colon carcinogenesis at a later stage. Transforming growth factor-beta (TGF-β) is a potent inhibitor of normal epithelial cell proliferation and is increasingly implicated in fibroid growth. Many tumor cell lines do not respond to the inhibitory effects of TGF-β due to a reduction or absence of the type II receptor (TGF-β R2) [45]. The down-regulation of TGF-β R2 in uterine fibroids is consistent with this finding. The PCP4 gene encoding PEP-19 is a calmodulin-regulatory protein found abundantly within neurons that was found to be increased in uterine fibroids. A study in PC12 cells [46] demonstrated that PEP-19 could inhibit apoptosis in the cells, suggesting that its up-regulation in human uterine fibroids may be similar. Thus, estrogen may promote cell survival and tumor growth by increasing expression of the anti-apoptotic PCP4 gene and by suppressing the expression of growth inhibitory receptors PTGER3 and TGF-β R2.

The calcitonin receptor-like receptor (CALCRL or CRLR), a G-protein coupled receptor, acts as a receptor for adrenomedullin (ADM) or calcitonin gene related peptide (CGRP) depending on which receptor activity modifying protein (RAMP) it partners with [47]. Using a rat uterine fibroid-derived cell line (ELT3), Thota and Yallampalli [34] demonstrated that expression of CALCRL and RAMP1 increased with progesterone and decreased with estrogen, consistent with what Helvering et al found in the array work (Table 3). Down-regulation of CALCRL expression in the tumor and in response to estrogen may implicate two important aspects of the tumor growth, that is, cell proliferation and angiogenesis.

L-PGDS has dual molecular functions. It catalyzes the conversion of prostaglandin H2 (PGH2) to prostaglandin D2 (PGD2) inside the cell and binds to small non-substrate lipophilic molecules including retinal, retinoic acid and thyroid hormone and serve as a transporter for these molecules once secreted [48]. ANXA1 is a calcium-dependent phospholipid binding protein and belongs to the annexin family. It was originally identified as a protein that apparently modulated the release of arachidonic acid from cells [49]. Recent data have shown that ANXA1 may specifically target cytosolic phospholipase A2 (PLA2) by both direct enzyme inhibition and suppression of cytokine-induced activation of the enzyme [50]. CRABP2 is a member of a large family of small proteins that specifically bind lipophilic compounds such as fatty acids and retinoids [28]. Recent work has suggested that CRABP2 may have a role in the movement of retinoic acid (RA) to the RA receptors (RARs), thereby enhancing the action of RA in the cells in which it is expressed. ALDH1A1 belongs to the aldehyde dehydrogenase family and is a terminal enzyme of the pathway catalyzing conversion of retinal to RA [51]. NR4A1 (encoded by Nur77 in mouse, NGFI-B in rat, and TR3 in human) is an immediate-early responsive orphan nuclear receptor whose expression is rapidly induced by a variety of extracellular stimuli including estrogen as evidenced here (Table 3). Using Northern blot RNA analysis Cicatiello et al [37] reported that NR4A1 was transiently activated in the uterus (up to 20-fold) within 30–120 min after treatment of adult ovariectomized rats with estrogen.

**Figure 4:**
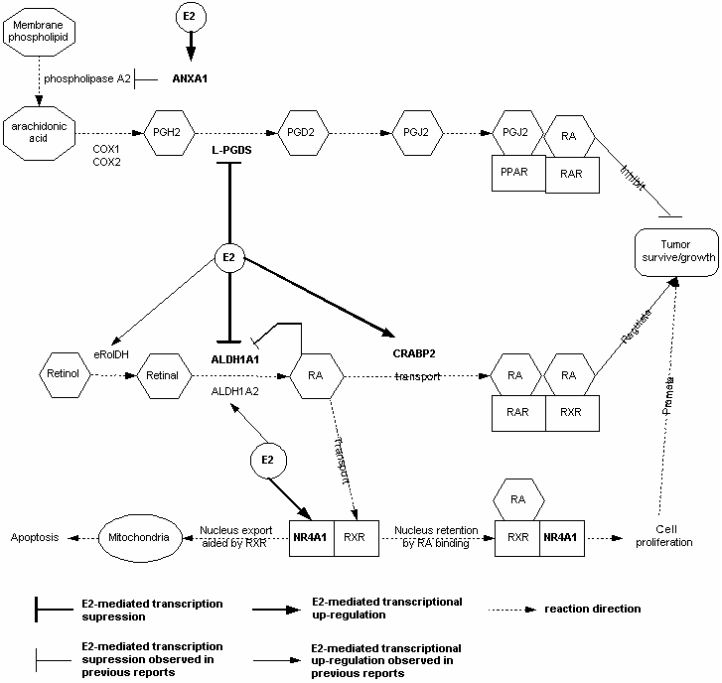
Multiple estrogen-regulated steps in the metabolisms of prostaglandin J2 and retinoic acid are implicated in estrogen-dependent growth of human uterine fibroids. Prostaglandin synthesis pathway was taken from the KEGG pathway database and simplified. Retinoic acid metabolic pathway was compiled based on previous work [51], [29]and [55]. E2 stands for 17 β-estradiol, PGH2 for prostaglandin H2, PGD2 for prostaglandin D2, PGJ2 for prostaglandin J2, RA for retinoic acid, RAR for RA receptor, RXR for retinoid × receptor, PPAR for peroxisome proliferator-activated receptor. Five human and rat ortholog and estrogen-regulated genes (NR4A1, ANXA1, L-PGDS, ALDH1A1 and CRABP2) are in bold font. NR4A1 is nuclear receptor subfamily 4, group A, member 1, which is also called NGFI-B (neuronal growth factor-induced clone B) in rat, Nur77 in mouse and TR3 in human. Its roles in cell proliferation and apoptosis summarized in the figure were largely taken from Cao et al [36] and references therein. ANXA1 stands for annexin A1, L-PGDS for lipocalin-type prostaglandin D2 synthase, ALDH1A1 and ALDH1A2 for aldehyde dehydrogenase 1 family, member A1 and member A2 respectively, CRABP2 for cellular retinoic acid binding protein 2, COX1 and COX2 for cyclooxygenase 1 and 2, eRolDH for epithelial retinal dehydrogenase [63].

Five of the ortholog genes (ANXA1, L-PGDS, ALDH1A1, CRABP2 and NR4A1) were found to be linked via their physiological functions following pathway analysis as summarized in Fig. 4. Estrogen may antagonize PPARγ mediated signaling first by up-regulating ANXA1 gene expression, thereby inhibiting phospholipase A2 activity and reducing arachinoic acid production, a common substrate for prostaglandin synthesis. Secondly, estrogen may antagonize PPARγ mediated signaling by down-regulating L-PGDS gene expression, which reduces conversion of PGH2 to PGD2 thus decreasing synthesis of PGJ2, an endogenous ligand for PPARγ activation. Recent work may provide clues to understand the physiological significance of these molecular events in the context of estrogen-dependent fibroid growth. Houston et al. [11] demonstrated that PPARγ upon its activation by its endogenous ligand PGJ2, suppressed estrogen-dependent fibroid growth. The authors hypothesized that PPARγ achieved its antagonistic effect by competitively binding to estrogen-responsive elements on promoters of genes which may be required for estrogen-dependent tumor growth. Yuan et al [52] showed that PPARγ activation by pioglitazone greatly retarded the progression of rat hepatic fibrosis. Shimada et al [53] reported that PPARγ ligands, including PGJ2, induced apoptosis in colon cancer cells and the same group [54] demonstrated that PPARγ plays an important role in the regulation of cell growth and cell death in gastric epithelial cells. It was reported [12] that human uterine fibroids expressed significantly higher levels of PPARγ, retinoid × receptor α and all-trans retinoic acid than myometrium. Thus we hypothesize that estrogen helps tumor cell survival and growth by interfering with PPARγ-mediated apoptosis at multiple steps in the biosynthesis of its ligand.

Retinoic acid (RA) has been established as a biologically active form of vitamin A (retinol). Fig 4 shows that biosynthesis of RA occurs by two sequential irreversible oxidations, first producing retinal from retinol by retinal dehydrogenases (RolDHs). The retinal is then oxidized to RA by retinal dehydrogenases (RalDHs) of the alcohol dehydrogenase (ADH) family 1 (also known as ALDH1A1-3). Tsibris et al [12] demonstrated that estrogen induced formation of abdominal fibroids in the guinea pig, and RA in combination with estrogen was required to specifically induce formation of fibroids in the uterus. Estrogen-induced guinea pig uterine fibroids are similar to the human fibroids, in that they also expressed increased levels of PPARγ, RXR-α and all-trans retinoic acid. Our integrated analysis of expression data collected by independent studies revealed that expression of two genes, ALDH1A1 and CRABP2 for RA synthesis or mobilization was differentially expressed in human uterine fibroids and regulated by estrogen in the rat uterus. Previous studies in rodents have suggested that estrogen directly induces uterine RA synthesis by increasing expression of epithelial retinal dehydrogenase (eRolDH) and ALDH1A2 (reviewed in [29]). Reduction of ALDH1A1 expression (Table 3) may not directly result from estrogen regulation but rather be due to feedback inhibition by RA [55]. RA acts on tumor growth by binding and regulating the tumor cell heterodimeric receptors RAR and RXR. RXR may also exert its effects on tumor cell survival and growth by partnering with PPARγ and/or NR4A1. NR4A1 was originally recognized for its role in cell proliferation and differentiation. It may confer its growth effects by trans-activating target genes required for cell proliferation in nucleus [56]. On the other hand, it also acts on mitochondria (non-trans-activation activity) to induce apoptosis. It was demonstrated that its nuclear export and initiation of NR4A1-dependent apoptosis depends on the nuclear exporting signal (NES) residing on its binding partner RXR and is suppressed by RA [36]. Thus, increased expression of NR4A1 and elevated levels of RA in response to estrogen could promote cell proliferation and suppress pro-apoptosis activity. Proteolipid and DM20 encoded by the PLP1 gene through alternative splicing are major structural components of central nervous system myelin. It has been well established that steroid hormones such as estrogen and progesterone regulate the expression of myelin proteins such as proteolipid protein (reviewed in [57]). However, what roles PLP1 may play in the development of human uterine fibroids needs further study. Similarly we have little knowledge of what functions that RNase A family, 4 (RNASE4) plays in the tumor growth. It is worthwhile to note that directions of expression changes of ANXA1, NR4A1 and PLP1 were opposite from that of the estrogen treated rat uterus.

Human genetics studies have shown that 40–50% of human uterine fibroids display karyotypically detectable chromosomal abnormalities [2]. Twenty percent of the abnormality is the characteristic translocation t(12:14) [58,59]. The twelve estrogen-regulated genes identified in the present study did not map to this translocation. The most prominent predisposition genes involved in human uterine fibroids, HGMI(c) and HMGI (Y), account for nearly 50% of the genetic variation in human uterine fibroids [59]. Dysregulation of these genes through chromosomal translation is a major event in uterine fibroid formation. Rearrangement of HMGI(C) and HMGI(Y) is also a very frequent event in many mesenchymal tumors, suggesting a critical role of the HMGI complex in tumorigenesis. The two key estrogen regulated pathways (e.g. RA and PPARγ) identified in the present study were both reported to be interacting with the HMG complex. For example, orchestrated action from PPARγ, HMGI(C), and other transcription factors is required in directing adipocyte differentiation [60]. Altered activity of these transcription factors could lead to biased differentiation of adipocytes or even adipocyte hyperplasia. Moreover, the retinoic acid pathway could regulate or be regulated by HMGI(C) during neuroblastoma tumorigenesis. Both HMGI(C), and HMGI(Y) are expressed in neuroblastoma cell lines and tumors and they are regulated by RA both at the RNA and protein levels, and can affect the responsiveness of these cells to RA [61,62]. A causal link has been proposed between HMGI(C) expression and RA induced growth arrest during tumorigenesis. Given the pivotal roles of the HMG complex in the fibroids and their interaction with PPARγ and RA pathways in controlling adipocyte growth and tumorigenesis of neuroblastoma, we speculate that estrogen may regulate fibroid growth through the PPARγ and RA pathways and their interaction with the HMGI(C) and HGMI(Y) complex. However, the direct link between HMG complex with RA and/or PPARγ in human uterine fibroids needs to be established experimentally, and is an area for future investigation.

## Conclusion

In conclusion, integration of multiple DNA microarray studies through ortholog gene analysis identified twelve uterine fibroid disease genes that may respond to estrogen in the fibroid. Functional and pathway analyses suggested multiple molecular mechanisms for estrogen-dependent growth of human uterine fibroids: enhanced tumor cell survival by increased expression of PCP4 and decreased expression of TGF-β R2 and PTGER3 and the complex interplay among five distinct nuclear receptors (ER, RAR, RXR, NR4A1 and PPARγ) that may enhance tumor cell survival and growth. Fully understanding the exact molecular interactions among these genes requires further study to validate their role in uterine fibroids. This work provides direction for studies which could influence the future direction of therapeutic intervention for the disease.

## Competing interests

The author(s) declare that they have no competing interests.

## Authors' contributions

TW, AGG, CS, JS and MNC participated in collecting, analyzing and interpreting array data. TW drafted the manuscript. HQ performed statistical analysis of the array data published by Hoffman et al (2003). LMH, NHK, HUB and JEO designed the rat uterus array study. JEO supervised the overall work. All authors read and approved the final manuscript.

## Pre-publication history

The pre-publication history for this paper can be accessed here:


